# Determination of Essential and Toxic Elements by ICP–MS in Herbal Medicines

**DOI:** 10.1021/acsomega.5c05971

**Published:** 2025-10-23

**Authors:** Juliana Naozuka, Aline Pereira de Oliveira, Higor Bolignano de Oliveira, Leon de Oliveira Lima, Cassiana Seimi Nomura

**Affiliations:** † Department of Chemistry, 28133 Federal University of Sao Paulo, 275 Prof. Artur Riedel Street, Diadema 09972-270, São Paulo, Brazil; ‡ Department of Fundamental Chemistry, Institute of Chemistry, University of Sao Paulo, 748 Prof. Lineu Prestes Av., São Paulo 05508-900, São Paulo, Brazil

## Abstract

This review synthesizes applications of inductively coupled plasma–mass spectrometry (ICP–MS) and hyphenated techniques for elemental determination in herbal medicines. The herbal medicine analysis has become increasingly important due to their widespread use in traditional and modern therapies. Due to the elements naturally present or contaminants (toxic elements) in herbal medicines and daily mineral requirements at the microgram level, analytical methods capable of precisely determining elemental concentrations in herbal medicines are essential. Analyses of several species have been conducted using ICP–MS, including rooibos (*Aspalathus linearis*), tea (*Camellia sinensis*), cannabis (*Cannabis sativa L.*), lemon (*Citrus limon*), turmeric (*Curcuma longa L.*), etc. The distribution of publications focused on different plant species from 2015 to 2025 is presented in this work. In studies focused on herbal medicine analysis using ICP–MS, sample preparation for elemental determination follows well-established methods, including closed-vessel acid digestion, direct infusion, and extraction of target compounds, among others. Beyond bulk elemental determination, coupling ICP–MS with other complementary analytical techniques, such as laser ablation inductively coupled plasma mass spectrometry (LA–ICP–MS), liquid chromatography–inductively coupled plasma–mass spectrometry (LC–ICP–MS), high-performance liquid chromatography–inductively coupled plasma–mass spectrometry (HPLC–ICP–MS), and flow injection–chemical vapor generation–inductively coupled plasma–mass spectrometry (FI–CVG–ICP–MS), among others, greatly enhances the analytical capabilities for the wide-ranging analysis of herbal medicines. As a result, these approaches facilitate a comprehensive understanding of the chemical, nutritional, and pharmacological properties of herbal medicines, improving quality control, safety assessment, and evaluation of their therapeutic potential.

## Graphical abstract

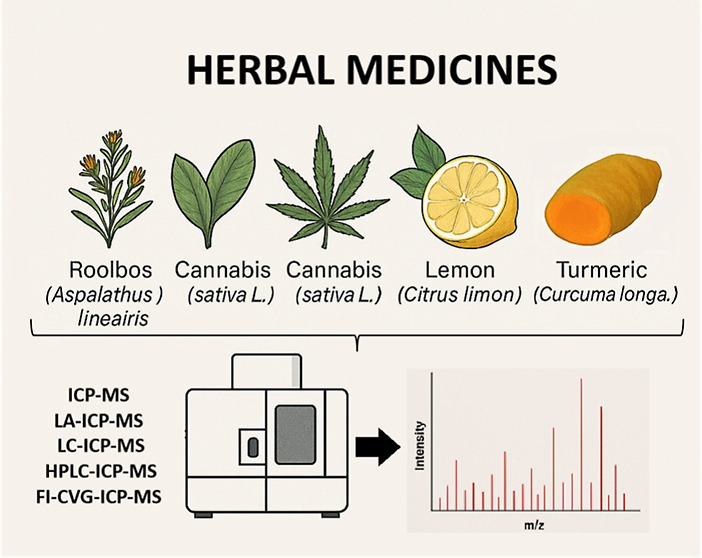

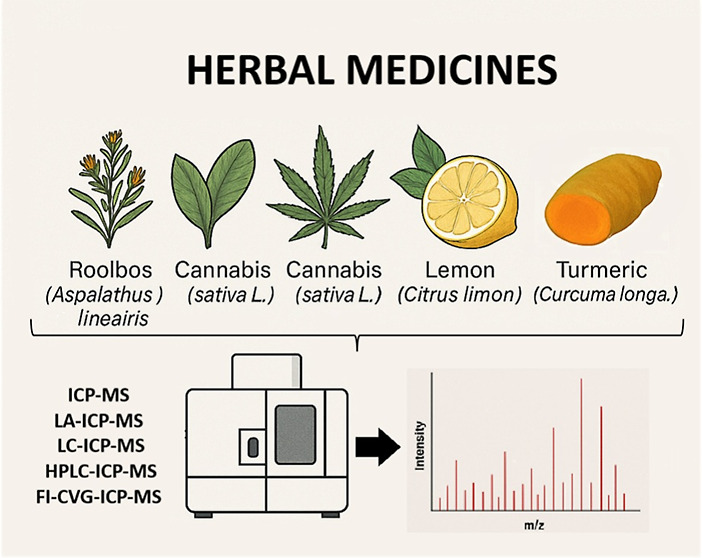

## 1 Introduction

Spectrometric techniques are widely used to elemental determination across several matrices, such as environmental, biological, food, and industrial matrices. Atomic fluorescence spectrometry (AFS), flame atomic absorption spectrometry (F AAS), graphite furnace atomic absorption spectrometry (GF AAS), ultraviolet–visible molecular absorption spectrophotometry (UV–vis), and inductively coupled plasma optical emission spectrometry (ICP OES) are successful and well-established techniques.^1^

Inductively coupled plasma mass spectrometry (ICP–MS) gained ground in solving analytical problems in the early 1980s, despite competition from established spectrometric techniques and its high cost initially limited adoption. However, the ability of the ICP–MS to determine trace and ultratrace element concentrations sparked scientific interest.^1,2^

Plant-derived materials, including roots and rhizomes, contain diverse therapeutic components. Traditional Chinese patent medicines, which use plants, animals, and minerals, are a significant source of therapeutic components.^3^ Essential and toxic elements are common in these medicines. Research into the composition and distribution of inorganic elements can help us to understand toxicity and pharmacology and inform the development of new therapeutic resources. Quadrupole ICP–MS can measure over 70 elements rapidly and continuously.^2,4^ This article reviews ICP–MS analysis of herbal medicines.

Unfortunately, the constituents of most herbal therapeutic remedies are not properly labeled. With the foregoing in mind, a flame photometer, which is simple, inexpensive, and rapid, and ICP–MS, which has a high degree of sensitivity and specificity, are commonly used to determine the elements in the herbal remedies. As complementary and alternative medicines, herbal remedies have grown in popularity in recent years. The concentrations of salt, potassium, and calcium significantly affect kidney and liver function, and blood pressure is crucial because it influences the glomerular filtration rate.

Therefore, this review aims to provide an updated and comprehensive overview of ICP–MS applications in herbal medicine analysis, addressing sample preparation, analytical strategies, and coupling to complementary techniques. The manuscript is structured as follows: Section 2 presents an overview of ICP–MS and related techniques; Section 3 surveys the literature on ICP–MS applications in herbal medicines; Section 4 describes sample preparation methods; Section 5 discusses ICP–MS applications in herbal medicines from an analytical perspective; Section 6 addresses hyphenated techniques; Section 7 presents perspectives and future trends; and Section 8 concludes the review.

## 2 Overview of ICP–MS and Related Techniques

ICP–MS combines the advantages of ICP OES with mass spectrometry (MS). In this technique, ions are generated in an argon plasma and quantified in the mass spectrometer according to their mass-to-charge ratio (*m*/*z*), which for singly charged ions corresponds to their atomic mass. Mass resolution, expressed as *m*/Δ*m* (where Δ*m* is the mass difference between adjacent peaks and m is the nominal mass), enables isotope discrimination and determination of natural abundances.^1–6^ This capability highlights the use of ICP–MS for isotopic analysis.^7^

Despite being a powerful multielement analytical technique with low detection limits, ICP–MS is subject to spectroscopic and nonspectroscopic interferences. Spectroscopic interferences include isobaric overlaps, polyatomic ions, and doubly charged ions, while nonspectroscopic effects arise from matrix-induced signal suppression or enhancement, affecting aerosol transport, ionization efficiency, or ion-beam transmission.^8^ Accordingly, strategies to minimize or eliminate these interferences are summarized below.

Liquid samples are typically digested to reduce matrix effects before nebulization into fine aerosols.^9^ Common nebulizers differ in efficiency and tolerance to dissolved solids, and spray chambers help to remove large droplets. Temperature control of the spray chamber can improve analyte stability, lower solvent load to the plasma, and reduce oxide formation.^10^ Once in plasma, the sample undergoes desolvation, vaporization, atomization, and ionization. The torch is aligned with the MS interface, and the plasma is sustained by radio frequency power applied to the load coil. The interface couples the atmospheric-pressure ICP to the high-vacuum MS using metallic cones with small orifices,^10,11^ allowing ion transmission while blocking particulates, neutrals, and photons.^12,13^ Ion lenses then focus the ion beam and further remove unwanted species.

In the mass analyzer, the ions are separated by *m*/*z*. Quadrupole systems are the most commonly used,^14^ while double-focusing magnetic sector and time-of-flight analyzers offer higher resolution or faster acquisition, respectively. Collision/reaction cell (CRC) technology, positioned before the analyzer, uses inert or reactive gases such as He or H_2_ to mitigate interferences.^15,16^ In ICP–MS/MS, an additional quadrupole improves control over precursor and product ions and avoids spectral overlap.^15^

Additional approaches extend ICP–MS performance: sector-field high-resolution ICP–MS (SF–ICP–MS) increases resolving power,^17–19^ while time-of-flight (TOF) ICP–MS acquires the full mass range quasi-simultaneously, enabling rapid, multielement detection.^20,21^ Laser ablation ICP–MS (LA–ICP–MS) allows direct solid analysis with minimal preparation, enabling in situ spatial analysis. Finally, for elemental chemical speciation, coupling separation techniques, mainly liquid chromatography, with ICP–MS (LC–ICP–MS) is essential. After fractionation, species concentrations are often far below the total concentration; thus, ICP–MS detection provides the sensitivity required for species-level quantification at trace or ultratrace levels, complementing bulk elemental data.^21–25,57^

## 3 Literature Overview: ICP–MS Applications in Herbal Medicines

The analysis of herbal medicines has become increasingly important due to their widespread use in traditional and modern therapies. These plants have a high concentration of phytochemical molecules (tannins, phenolic compounds, alkaloids, and flavonoids), and their extracts have been used to treat diseases, but there is the possibility of chronic adverse effects on human health, and it is fundamental to determine trace elements and their species in herbal preparations aiming to optimize their therapeutic efficacy.^6^

Analyses of several species have been conducted using ICP–MS, including rooibos (*Aspalathus linearis*), tea (*Camellia sinensis*), cannabis (*Cannabis sativa L.*), lemon (*Citrus limon*), turmeric (*Curcuma longa L.*), fennel (*Foeniculum vulgare*), hibiscus (*Hibiscus sabdariffa*), elderberry (*Sambucus nigra L.*), chamomile (*Matricaria chamomilla*), German chamomile (*Matricaria recutita L.*), lemon balm (*Melissa officinalis L.*), peppermint (*Mentha piperita L.*), olive (*Olea europaea L.*), American ginseng (*Panax quinquefolius L.*), guarana (*Paullinia cupana L.*), castor bean (*Ricinus communis L.*), sage (*Salvia officinalis L.*), sesame (*Sesamum indicum*), chirata (*Swertia chirayita*), and mung bean (*Vigna radiata*).^3,26–29,50^

Considering the publication trends, the largest proportion of publications indexed in current databases over the last 10 years for medicinal plant analysis reports are in the disciplines of pharmacology and pharmacy (Figure 1). Figure 1 presents the distribution of publications related to different plant species in the period from 2015 to 2025. The figure consists of a pie chart illustrating the relative percentages of publications corresponding to each herbal medicine based on data retrieved from the Web of Science database. The search used keyword combinations such as “herbal medicine” or “medicinal plants” or “ phytotherapeutic” combined with “mass spectrometry”. To refine the results and identify which species were most frequently investigated by mass spectrometry, additional research combined the terms already mentioned with the scientific names of each medicinal plant. With this approach, it was possible to obtain a more accurate estimate of publication frequency for each species over the ten-year period. Finally, the chart was generated in Microsoft Excel to provide a clear and accessible view of the publication trends.

**Figure 1:**
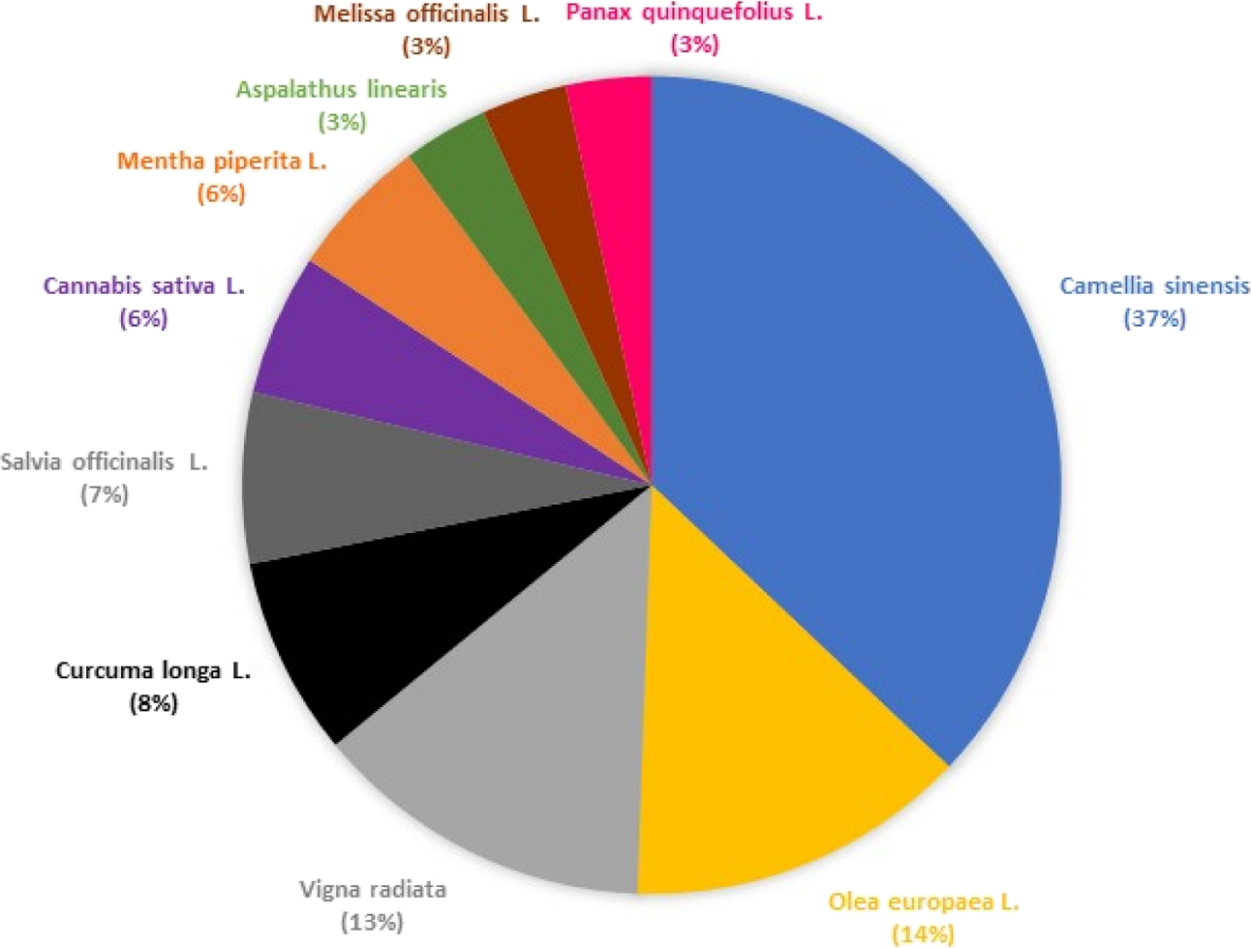
Medicinal plants in herbal medicine publications utilize mass spectrometry as the predominant technique. Authors’ compilation based on WoS data.

ICP–MS has emerged as a powerful analytical technique for the determination of trace and ultratrace elements in herbal medicines due to its high sensitivity and ability to detect low concentrations. ICP–MS plays a crucial role in ensuring the safety, quality, and efficacy of these products. The growing interest in natural health products, combined with the high sensitivity, robustness, and versatility of ICP–MS for coupling with other analytical techniques, makes it an essential tool in quality control and regulatory compliance. In this context, it is particularly valuable to identify and quantify contaminants, e.g., potentially toxic elements such as As, Al, Ba, Cd, Ni, Pb, and Sb,^4,19,30^ and to access the nutritional profile of herbal formulations.^31,32^ Regarding the analytes targeted in these studies, it has been observed that the descending order of publication frequency is as follows: As and Mn > Cu and Fe > Co and Pb > Al, Ca, K, Li, Mg, and Zn, in addition to metabolites,^29,33^ with other elements being addressed in fewer publications.

Accordingly, the analysis of herbal medicines by ICP–MS has gained interest, as reflected in the publication numbers from 2015 to 2025, shown in Figure 2. Figure 2 presents a bar chart illustrating the number of publications in each year, considering the total publication in an 11 year period. The graph was produced by the authors using Microsoft Excel, based on data retrieved from the Web of Science database. The search was conducted using the keywords “herbal medicine” or “phytotherapeutic” or “medicinal plants”, combined with “ICP–MS”, with the raw data subsequently organized by publication year. The low number of publications recorded for 2025 reflects the limited data available at the time the search was performed as of January 20, 2025.

**Figure 2:**
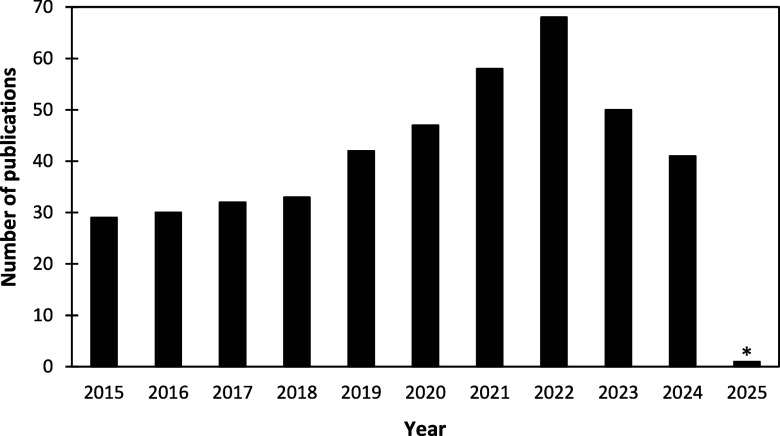
Publication numbers from the years 2015 to 2025 using “ICP–MS” and “herbal medicine” or “phytotherapeutic” or “medicinal plants” as keywords. Authors’ compilation based on WoS data. *Publications until January 2025.

Additionally, the most significant increase in publications occurred between 2015 and 2022 (Figure 2). The observed increase in research related to medicinal plants during the pandemic period (2020–2022) can be attributed to the heightened interest in their potential as supplements. This trend is further supported by the noticeable rise in studies on compounds such as quercetin, curcumin, Andrographis paniculata, tannins, and flavonoids.^3,28^ Between 2015 and 2018, annual publications on these topics averaged approximately 3,650, with a significant increase to approximately 7,380 between 2019 and 2022, according to the Web of Science database. Quercetin, a flavonoid present in various plants, has demonstrated potential in inhibiting several respiratory infections.^34^ Curcumin, found in turmeric (*C. longa*) and its constituents like cyclocurcumin, has recently shown promise in binding to the active site of the main protease of SARS-CoV-2.^35^ Similarly, Indian ginseng (*A. paniculata*), a plant widely used in Asian medicine and containing compounds such as andrographolide and dihydroxy dimethoxyflavone, has exhibited binding properties comparable to those of curcumin.^36^

However, studies focused on medicinal plants face several key challenges. These challenges are particularly evident when detecting and analyzing potentially toxic elements, which may arise from environmental factors, contaminants, or plant preparation methods. Such plants may contain bioactive compounds, and many are used in polyherbal formulas, which often contain a complex mixture of multiple plant species. ICP–MS–based elemental determination in herbal medicines also presents several challenges, including often complex sample preparation, low concentrations of trace elements, detection limit constraints, and matrix effects in multielement analyses, all of which can affect the precision and reliability of the analytical results. Furthermore, plant samples often exhibit variability in their elemental composition due to factors, such as geographic location, growth conditions, and harvesting methods, which may complicate data interpretation.

Beyond elemental determination through bulk analysis, coupling ICP–MS with other complementary analytical techniques, such as LA–ICP–MS, LC–ICP–MS, high-performance liquid chromatography (HPLC–ICP–MS), and flow injection cold vapor generation (FI–CVG–ICP–MS), among others, greatly enhances the analytical capabilities for the wide-ranging analysis of herbal medicines. These coupled techniques enable elemental determination, high-resolution profiling of elemental composition, and chemical speciation analysis. As a result, they facilitate a comprehensive understanding of the chemical, nutritional, and pharmacological properties of herbal medicines, improving their quality control, safety assessment, and evaluation of their therapeutic potential.

Finally, scientific publications from 2015 to 2025 are summarized in Tables 1–3, which compile ICP–MS–based applications in herbal medicines—covering bulk ICP–MS, LC/SEC–ICP–MS (speciation), and LA–ICP–MS—alongside the corresponding sample preparation methods.^3,26–31,33,37–49^

**Table 1:** Bulk ICP–MS in Herbal Matrices (Solution/Infusion)

herbal medicine	analytical technique	analyte	sample preparation	ref
*Aspalathus linearis*; *Camellia sinensis* L.; *Foeniculum vulgare*; *Hibiscus sabdariffa* L.;*Matricaria camomilla*; *Mentha piperita*; *Sambucus nigra*	ICP–MS	Al, As, Cd, Cu, Hg, and Pb	dried samples and infusions	^26^
*Cannabis sativa* L.	ICP–MS	Fe	acid digestion in a closed microwave system for bulk analysis	^29^
*Citrus limon*	ICP–MS	Ag, Al, As, Ba, Bi, Co, Cr, Cu, Fe, Ga, In, La, Li, Mn, Mo, Ni, Rb, Sb, Sc, Se, Sn, Sr, Tl, V, and Zn	acid digestion in a closed microwave system for bulk analysis	^39^
*Curcuma longa* L.	ICP–MS	B	acid digestion in a closed microwave system for bulk analysis	^28^
*Mentha piperita* L.	ICP–MS	Ag, Au, Co, Cs, Li, Mo, Se, Sr, and V	infusion	^41^
*Mentha piperita* L.	ICP–MS	As, Cd, and Pb	infusion	^42^
*Olea europaea* L.	ICP–MS	Al, As, B, Ba, Ca, Co, Cr, Cu, Fe, K, Li, Mg, Mn, Na, Ni, Pb, Sr, and Zn	acid digestion in a closed microwave system for bulk analysis	^43^
*Olea europaea* L.	ICP–MS	Al, As, Ba, Cd, Co, Cu Cr, Fe, Mn, Ni, Pb, Se, Sr, and Zn	infusion	^27^
*Olea europaea* L.	ICP–MS	Ca, Cu, Fe, K, Mg, Mn, and Na	acid digestion in a closed microwave system for bulk analysis	^30^
*Panax quinquefolius* L.	ICP–MS	B, Ca, Cu, Fe, K, Mg, Mn, Mo, Na, P, and Zn	drying, pulverization, and acid digestion (bulk analysis)	^44^
*Ricinus communis* L.	ICP–MS	As, Ca, Cu, Fe, K, Mg, Mn, N, P, and Zn	drying, grinding, and acid digestion in a closed microwave system	^45^
*Ricinus communis* L.; *Foeniculum vulgare*; *Vigna radiata*; *Sesamum indicum*	ICP–MS	Al, Ag, Ba, Bi, Cd Co, Cr, Cu, Hg, Mn, Mo, Ni, Pb, and Zn	acid digestion in a closed microwave system	^3^
*Sambucus nigra* L.	ICP–MS	Ag, As, Be, Bi, Cd, Co, Cs, Ga, Hg, In, Li, Ni, Pb, Rb, Tl, and U	acid digestion in a closed microwave system	^31^
*Swertia chirayita*	ICP–MS	Li, Na, Mg, Al, Si, K, Ca, Sr, Ba, Ti, Mn, Fe, and Cr	collection, sun-drying, comminution, and acid digestion	^46^

**2:** LC/SEC–ICP–MS (Speciation) and Hyphenated ICP–MS Studies in Herbal Matrices

herbal medicine	analytical technique	analyte	sample preparation	reference
*Calligonum comosum*, *Citrullus colocynthis*, *Haloxylon salicornicum*, *Momordica charantia*, *Nigella sativa*, *Olea europaea*, *Opuntia ficus-indica*, *Pennisetum glaucum*, *Sesamum indicum*, *Trigonella foenum-graecum*, *Vaccinium myrtillus*	SEC–ICP–MS	Zn	ground dry samples and water extraction	^37^
*Calligonum comosum*, *Citrullus colocynthis*, *Haloxylon salicornicum*, *Momordica charantia*, *Nigella sativa*, *Olea europaea*, *Opuntia ficus-indica*, *Pennisetum glaucum*, *Sesamum indicum*, *Trigonella foenum-graecum*, V*accinium myrtillus*	ICP–MS and SEC–ICP–MS	Cr	acid digestion in a closed microwave system for bulk analysis and ground dry samples and water extraction for speciation	^38^
*Matricaria recutita* L.; Mentha x piperita; *Melissa officinalis* L.; *Salvia officinalis* L.	ICP–MS and HPLC–ICP–MS	As and arsenic species (As(III), As(V), AB, MMA, and DMA)	acid digestion in a closed microwave system for bulk analysis and extraction using water/methanol solution (1:9 ratio) for speciation	^33^
*Maytenus ilicifolia*, *Passiflora incamata* L, *Paullinia cupana*, *Peumus boldus*	ICP–MS and FI-CVG-ICP–MS	As, Cd, and Pb (ICP–MS); As, and Hg (FI-CVG-ICP–MS)	acid digestion in a closed microwave system and dry ashing	^40^

**Table 3:** LA–ICP–MS Studies in Herbal Medicine Plant Tissues

herbal medicine	analyte	solid sample preparation	main finding	ref.
*Triteleia peduncularis* (leaf sections)	Zn, Cu, Sr, Mn	cryosectioned tissue; section thickness kept constant (e.g., 10–50 μm); supported on adhesive; preablation/scouting to remove surface contamination	distinct spatial distributions (Zn gradient from veins to edges; Cu uniform with higher base; Sr in petiole; Mn enriched in main vein)	^47,48^
*Coptis chinensis* Franch. (rhizome sections)	Cd (with Ca, P correlation)	sectioned rhizomes; geometry/flatness controlled for ablation	preferential Cd accumulation in periderm and cortex; outer regions implicated in accumulation	^45^
*Star anise*; passion fruit (solid samples)	As, Cd, Co, Cr, Cu, Mn, Ni, Pb, Sr, V, and Zn	bulk comminution; pressed/flat sample surface for spot/line ablation	feasibility of multielement quantification in plant-derived solids using matrix-simulated calibration	^48,49^

## 4 Sample Preparation Methods

In studies focused on the analysis of herbal medicines using ICP–MS, sample preparation for elemental determination typically follows well-established methods, including closed-vessel acid digestion,^31,43^ direct infusion,^27^ and extraction of target analytes,^33^ among others. In addition, rigorous control of contamination sources (e.g., reagent purity, vessel cleanliness, laboratory environment) and of total dissolved solids (TDS) introduced into the plasma is essential to ensure accuracy and robustness in herbal matrices.^9^ To support reproducibility, independent preparation replicates (*n* ≥ 3) are performed, procedural blanks and plant-based CRMs (certified reference materials) are included, intra/inter-day RSDs of ≤5–10% for major/minor elements and ≤15% at ultratrace levels are targeted, and postdigestion TDS ≤0.2% w/v is maintained to preserve plasma robustness.^8,9^

In general, elemental determination in medicinal plants typically uses closed-vessel microwave-assisted digestion using oxidizing mixtures, predominantly composed of dilute HNO_3_ and H_2_O_2_, to effectively digest the organic matrix of the sample.^40^ This method ensures efficient digestion of the plant matrix by releasing the constituent elements in their inorganic form into solution. To minimize blanks and memory effects, high-purity reagents (e.g., sub-boiled HNO_3_) and ultrapure water should be used, and PTFE or quartz vessels should be precleaned.^9^ Recommended sample intakes are commonly 200–500 mg of well-homogenized plant material per vessel to balance representativeness and digestion load; when higher masses are needed (e.g., for ultratrace targets), proportional reagent volumes and longer ramps help avoid incomplete oxidation or overpressurization.^9,40^ Typical programs reach 180–220 °C with staged ramps/holds to prevent venting losses of volatile species and to complete oxidation of residual carbon.^9,40^ Postdigestion, maintaining TDS ≤ 0.2% m/v (often via dilution) helps preserve plasma robustness and reduces matrix suppression.^9^ Internal standardization (e.g., In, Rh, Ir) and, when appropriate, collision/reaction cell operation mitigate matrix-driven signal drift and spectral overlaps.^8,15,16^ For interlaboratory comparability, digestion programs (ramp/hold temperatures and times), sample/reagent loads, and postdigestion dilutions are reported together with the chosen internal standards and collision/reaction cell settings. In this way, precise and accurate elemental determination in plant samples using ICP–MS and liquid sample introduction systems may be carried out, achieving limits of detection (LOD) and quantification (LOQ) at the parts-*per*-trillion level.^32,43^ Microwave-assisted acid digestion has been widely applied to herbal medicines, typically to plant-based samples such as leaves and flowers from different species.^3,45^ Muller et al. (2015)^40^ developed a microwave-assisted digestion method using dilute nitric acid (4 mol L^–1^) to determine As, Cd, and Pb in medicinal plants, following the United States Pharmacopeia Chapter 2232 (Elemental Contaminants in Dietary Supplements) guidelines. The method demonstrated efficient digestion with recoveries of 96–103% relative to CRMl values and allowed up to eight samples to be processed simultaneously, minimizing reagent use and waste generation, in line with green chemistry principles. The digestion process resulted in digests with carbon content below 320 mg L^–1^, ensuring minimal interference in subsequent analyses. For comparison, samples of *Passiflora incamata L.*, *P. cupana*, *Maytenus ilicifolia*, and *Peumus boldus* (up to 500 mg) were digested using HNO_3_ solutions (2–14.4 mol L^–1^) and the dry-ashing method recommended by pharmacopeias, with As, Cd, and Pb quantified by ICP–MS.^40^ When dry ashing is used, reagent and environmental blanks are particularly important due to higher contamination risks and potential volatilization losses (e.g., As, Hg).^9,40^

Sample preparation methods may include drying steps such as oven drying at controlled temperatures to prevent the loss of volatile elements while simultaneously removing moisture, as well as freeze-drying, thereby preserving the integrity of the samples, improving the accuracy of elemental determination, and minimizing interferences in direct solid sampling using laser-based approaches.^31^ Additionally, grinding steps with cryogenic mills, knife mills, or mortars and pestles are crucial to ensure sample homogenization, minimizing compositional variations in plant tissues before digestion and extraction.^31^ To maximize representativeness, primary samples should be subsampled after thorough homogenization (e.g., ball-mill or bead-mill) and, when feasible, prepared in replicates.^9^ Contact materials (steel jars, blades) must be checked for contribution of Fe, Cr, Ni, etc.; using ZrO_2_ or agate accessories can reduce carry-over of metallic contaminants.^50^ When steel accessories are unavoidable, potential Fe, Cr, and Ni contributions are tracked via blanks and fortified controls, and blank subtraction is applied where appropriate.

In infusion analysis, liquid samples are prepared from dried leaves following standardized methods that simulate household preparation.^26^ Critical variables include plant mass-to-water ratio, water temperature, infusion time, and filtration, all of which influence extraction yields and apparent element concentrations; these parameters should be reported to enable comparison across studies.^26,27^ These steps are often followed by comminution processes, such as grinding and sieving, to reduce particle size, thereby increasing the surface area available to reagents during acid digestion or extraction procedures.^40^ It is essential for improving analytical reproducibility and reducing variability in extraction yields. For instance, grinding and infusion have been applied to herbal medicines commonly consumed as tea, such as *M. piperita*.^41^ In studies focused on chemical speciation, efficient extraction that preserves the integrity of different chemical forms is commonly required, which can be performed using methods such as enzymatic digestion, water, a water/methanol mixture, or other extractants.^33^ For speciation, redox-state preservation (low temperature, oxygen control when relevant) and minimal sample handling are recommended; protein-binding or low-molecular-mass fractions may be isolated by SEC or ultrafiltration prior to ICP–MS detection.^24,25,37,38^ Filtration (e.g., 0.22 or 0.45 μm) and immediate analysis or cold storage help reduce interconversion.^33^ Time-course stability tests (e.g., at 4 °C and room temperature) are also performed to rule out species interconversion during storage and handling.

The study by Qazimi et al. (2019)^32^ assessed the response of chamomile, peppermint, lemon balm, and sage to elevated inorganic As levels in soil. A pot experiment was conducted with soils contaminated with As­(III) and As­(V), and As species, including As­(III), As­(V), arsenobetaine (AB), monomethylarsonic acid (MMA), and dimethylarsinic acid (DMA), were separated and quantified using HPLC–ICP–MS. To this end, tea samples were prepared by extracting 1.0 g of plant material for extraction with a 1:9 water/methanol solution, followed by HPLC–ICP–MS analysis for As speciation. This workflow highlights the need to validate extraction efficiency and to check for species stability during sample workup.^24,25,33^ Alternative methods, such as the infusions of medicinal plants, based on traditional knowledge, are used to assess the release of elements into aqueous solutions, simulating human consumption.^26,27^

For the direct analysis of the solid, proper sample preparation for LA–ICP–MS is crucial to ensure accuracy and reproducibility. LA–ICP–MS offers high sensitivity and spatial resolution, allowing direct solid analysis and trace-element detection in plant tissues. However, to fully exploit the technique’s potential, sample preparation must be meticulously planned and executed. The process involves several interdependent steps, each with specific challenges that must be appropriately addressed to optimize the analysis. Since LA–ICP–MS provides spatially resolved information, mass spectrometry imaging can be used in studies focused on medicinal plant analysis. This type of analysis requires specific precautions to preserve the native tissue structure and the integrity of the spatial elemental distribution. Accordingly, after sample collection and washing to remove surface contaminants, drying must be carefully controlled to minimize structural alterations. Plant parts such as roots, stems, and leaves are often supported on an adhesive tape^47^ to minimize surface deformation; variations in sample topography can affect laser focusing and compromise the analysis. For high-resolution analysis of different plant tissues, thin-section preparation is generally preferred and can be achieved by cryosectioning.^51^ Section thickness (e.g., 10–50 μm) should be consistent across samples to harmonize ablation rates; substrates and adhesives must be screened for elemental background to avoid false positives.^23,47,48^ Before imaging, preablation or low-fluence scouting lines can remove surface contamination; quantitative imaging benefits from matrix-matched microdroplet or solid standards and internal standardization to ^13^C or major elements.^23,47,53,54^ Instrumental conditions for LA–ICP–MS (fluence, spot size, scan speed) are kept constant across runs, and repeatability is assessed by replicate lines and/or areas with RSDs reported for key analytes.

For bulk analysis via direct solid sampling, sample preparation follows a different methodology. After drying, the sample must be comminuted and homogenized to minimize compositional variations between plant regions. Comminution is often performed using a knife or ball mill. An alternative to these mills is a cell-disruption system operating on the same principle as ball mills (bead mills). This homogenizer was first used by Zhou et al. (2022)^50^ and later applied by Cui et al. (2023)^47^ for the analysis of *Triteleia peduncularis*, achieving particle sizes <10 μm in 120 s. This system uses Zr beads, ensuring efficient grinding by disrupting plant cell walls while minimizing external Fe contamination, which can occur with other milling methods. Once particle size has been reduced to a fine, homogeneous powder, the next step is pelletization, in which the pulverized material is compacted under high pressure using a hydraulic press to form solid pellets. This approach mitigates challenges associated with direct solid analysis by LA–ICP–MS, particularly related to sample surface uniformity and matrix heterogeneity. Pellet formation ensures a flat, homogeneous surface, which is essential for laser–material interaction, reducing variation in ablation rate. In plant samples, tissue morphology can cause irregular ablation and inconsistent signals, but compacted pellets help mitigate this issue by reducing matrix heterogeneity through comminution and pelletization.^52^ When binders are required to improve pellet integrity, low-background cellulose or PTFE should be evaluated and blank-corrected; pressing pressure and dwell time should be standardized to improve reproducibility of ablation yield.^52^ Pellet density and surface roughness should be monitored to reduce the ablation-rate variability and improve quantitative consistency.

Quality assurance and validation considerations apply across all workflows: use of procedural blanks, replicate preparations, spike-recovery tests, and analysis of appropriate plant-based CRMs to verify trueness; reporting of LOD/LOQ calculation approach; and, for speciation, demonstration of species-specific recovery and absence of interconversion.^9,24,25,32,40^ Documenting these parameters enhances comparability between studies and strengthens conclusions on the safety and effectiveness of herbal products. Together with explicit precision targets and replicate designs, these QA/QC measures allow within- and between-laboratory reproducibility to be critically assessed.

## 5 ICP–MS Applications in Herbal Medicines through Analytical Approaches

This analytical technique is predominantly used to bulk analysis in most studies published in the literature on herbal medicines. It allows accurate determination of essential elements, such as Ca, Mg, and Fe, as well as potentially toxic elements like As and Cd in different parts of the plant tissues.^32,43^

Medicinal plants used in traditional antidiabetes treatment, such as *S. indicum*, *O. europaea*, *Vaccinium myrtillus*, *Nigella sativa*, *Trigonella foenum-graecum*, and others, have already been the focus of studies aimed at determining the total Cr concentration, as it has been reported that trace elements, such as Cr and Mn, positively affect diabetes mellitus.^38^ The total Cr contents were determined by SF–ICP–MS in samples previously subjected to acid digestion. Internal standards (Rh and Ir) with a concentration of 1 μg L^–1^ were added to each sample, and isotopes ^103^Rh and ^193^Ir were monitored. The instrument was operated in high-resolution mode, monitoring elemental isotope ^52^Cr along with the internal standards. The examined plants exhibited a wide range of total Cr contents (45–1880 μg kg^–1^). Furthermore, although Cr is considered a carcinogenic element, no cancer risk was identified from the consumption of any of the plants studied.^38^ *S. chirayita*, a prominent medicinal plant known for its therapeutic properties, was the subject of a compositional analysis conducted by Turan et al. (2024).^43^ The study evaluated different parts of the plant, including the root, stem, leaves, and flowers, using laser-induced breakdown spectroscopy (LIBS) and ICP–MS. The analysis quantified several elements, such as Li, Na, Mg, Al, Si, K, Ca, Sr, Ba, Ti, Mn, Fe, and Cr, offering valuable insights into the plant’s elemental composition.^46^

In a recent study by de Oliveira et al. (2024),^31^ ICP–MS was employed to determine the mineral composition of leaves and flowers of wild-grown *S. nigra* collected from 11 different locations in Kosovo. For this, 16 isotopes (^7^Li, ^9^Be, ^59^Co, ^60^Ni, ^79^Ga, ^75^As, ^85^Rb, ^107^Ag, ^114^Cd, ^115^In, ^133^Cs, ^202^Hg, ^205^Tl, ^209^Bi, ^238^U , ^206^Pb, ^207^Pb, and ^208^Pb) were monitored using ICP–MS. External calibration was used to elemental determination in the samples previously subjected to acid digestion. Detector attenuation mode was used to extend the linearity range without collision/reaction gas. In addition, He was used as a collision gas to determine Ni and Se by monitoring the isotopes ^60^Ni and ^78^Se. The leaves showed higher mineral content than the flowers, with significant geographic variations observed in both. Multivariate analysis identified distinct patterns in element concentrations, with specific elements being important for discriminating leaf and flower samples.^32^

ICP–MS has been applied to determine the elemental abundance in medicinal plants commonly used in the Kingdom of Saudi Arabia to treat various ailments in the form of folk medicine.^3^ To this end, forty-four medicinal plant samples were collected from local markets and evaluated for the presence of 14 elements: Mn, Cr, Co, Ni, Cu, Mo, Al, Pb, Ba, Zn, Ag, Hg, Bi, and Cd. Multielement detection was performed using a triple quadrupole ICP–MS system. The LODs ranged from 0.01 to 0.7 μg kg^–1^, whereas the LOQ ranged from 0.03 to 2.31 μg kg^–1^. Hg and Cd levels exceeded the maximum allowed limits in medicinal plants, while the other elements were within the acceptable range. Several plants showed high levels of these elements, such as *F. vulgare* (Pb, Hg, and Cd), *R. communis* (Pb and Cd), *V. radiata* (Pb and Cd), and *S. indicum* (Pb and Hg). The findings provide baseline data for comparative analysis of these medicinal plants, helping to select safe plants for consumer use and treatment of various ailments.^3^ Different varieties of olive leaves (*O. europaea*) have been studied to determine their elemental composition using ICP–MS.^31^ Accordingly, an elemental assessment of mature leaves and leaf sprouts was conducted by screening for the presence of 26 elements in previously digested plant tissues. External calibration was performed using a multielement standard solution, and In (10 μg L^–1^) was added online as the internal standard. The findings of the analysis allowed the authors to propose olive leaf extract from different cultivars as a feasible and economical source of elemental substrates for mitigating deficiencies in essential elements, including Na, K, Mg, Ca, Mn, Fe, and Cu.^31^ The effects of exposing medicinal plants to nanoparticles were studied. Deng et al. (2022)^29^ investigated the effect of iron oxide nanoparticles (Fe_3_O_4_ NPs) on the phenotype and metabolite changes in hemp (*Cannabis sativa L.*), an annual crop distributed worldwide. In this study, the plants were hydroponically grown in media supplemented with different concentrations of Fe_3_O_4_ NPs. ICP–MS was then used to determine the absorption and translocation of Fe from the nanoparticles by analyzing different plant parts (stem, leaf, and root), which were previously subjected to acid digestion. Plant uptake and translocation of Fe increased with the Fe_3_O_4_ NP concentration, reaching the highest level at 200 mg L^–1^. However, plants exposed to 500 mg L^–1^ showed lower Fe content, suggesting saturation at concentrations above 200 mg L^–1^.^29^

Other studies have evaluated the bioaccumulation of potentially toxic elements in combination with other environmental pollutants in herbal medicines using ICP–MS analysis. In this context, Turan et al. (2011)^43^ assessed the feasibility of using olive tree leaves (*O. europaea L.*) as a bioindicator for environmental pollution. To do so, leaf samples were used to estimate the pollution level by measuring the concentrations of Al, As, B, Ba, Ca, Co, Cr, Cu, Fe, K, Li, Mg, Mn, Na, Ni, Pb, Sr, and Zn by using ICP–MS and calculating the pollution factor values. A single quadrupole ICP–MS was employed for elemental analysis, and five-level external calibration was performed for each element to ensure accurate quantification, with acceptable calibration curves (*R* ^2^ > 0.99). Helium was used as a collision gas, and H_2_ was a reaction gas. Olive leaves have proven to be reliable bioindicators of pollution in the Mediterranean, where they grow naturally and are cultivated.^43^ The mobility, translocation, and bioaccumulation of As using ICP–MS-based analyses have also been assessed in herbs such as chamomile (*M. recutita (L.) Rauschert*), peppermint (*Mentha x piperita*), lemon balm (*M. officinalis L.*), and sage (*S. officinalis L.*).^33^ To this end, the experiments were conducted in soils artificially contaminated with As­(III) and As­(V). ICP–MS was applied to determine the As content in the soil and plants, and the ability of the herbs to accumulate As was subsequently assessed. The results indicated that the oxidized form of As had a higher capacity for accumulation in herbs and was more easily absorbed by plants from the substrate.^33^ Bioaccumulation of As in medicinal plants has also been studied in castor plants (*R. communis L.*).^45^ Arsenic levels were quantified using ICP–MS. The translocation factor (TF) and bioconcentration factor (BCF) were determined, with TF values ranging from 0.20 to 0.63 and BCF values between 0.28 and 0.75.^45^ Determination of toxic elements (As, Cd, and Pb) in medicinal plants (*P. incamata L.*, *P. cupana*, *M. ilicifolia*, and *P. boldus*) by ICP–MS was reported by Muller et al. (2015).^40^ External calibration was performed, and the following isotopes were monitored: ^75^As, ^111^Cd, and ^208^Pb.

Evaluation of adulteration in products derived from medicinal plants has been carried out by ICP–MS, such as the assessment of *C. longa* extract for adulteration with synthetic curcumin reported by Girme et al. (2020).^28^ In this way, the presence of B was determined as a qualitative indicator, as it originates from the synthetic curcumin production process. Synthetic curcumin extracts showed B levels greater than 250 mg kg^–1^, while natural-origin extracts contained less than 2.0 mg kg^–1^.^28^ As orthogonal confirmation (beyond ICP–MS), LC–MS profiling of curcuminoid ratios (curcumin/demethoxycurcumin/bisdemethoxycurcumin) and radiocarbon (^14^C) measurements are commonly used to distinguish synthetic from plant-derived curcumin;^53^ within ICP–MS workflows, screening for Pb/Cr signatures compatible with lead chromate adulteration in turmeric powders can flag economic adulteration, although this finding alone is not diagnostic of synthetic origin.^54^ In addition to studies focused on the adulteration of medicinal plants, ICP–MS-based methodologies have been applied to trace the geographical origin of these plants based on their trace element profiles. American ginseng (*P. quinquefolius L.*) was the subject of studies conducted by Shuai et al. (2023), who carried out elemental characterization and stable carbon and nitrogen isotope analysis to determine its geographical origin combined with chemometrics. External calibration was applied to determine the following elements in the plants, which were previously ground and subjected to acid digestion: K, Ca, Na, Mg, P, Fe, Mn, Al, Ba, Zn, B, Rb, Ni, Sr, Cu, Pb, Cr, As, Se, Cd, Mo, Be, Cs, V, Co, and Sn; Ge, Bi, Li, Y, In, and Sc were used as internal standards. It was reported that the combination of multielement analysis and carbon/nitrogen stable isotope ratios, along with chemometric models like support vector machine, linear discriminant analysis, random forest, and feedforward neural network, successfully classified American ginseng from four different regions. The feedforward neural network model achieved perfect accuracy (100%), highlighting the usefulness of these elements and isotopes in determining the geographical origin of American ginseng.^44^

Medicinal plant infusions, grounded in traditional knowledge, have been analyzed using ICP–MS to evaluate the release of elements into liquid solutions, simulating human consumption for trace element assessment. Contents of potentially toxic elements (As, Pb, and Cd) in mint tea (*M. piperita L.*) infusions available in Polish markets were evaluated by Huang et al. (2023b).^51^ The investigated mint tea infusions showed low levels of potentially toxic elements, as follows: As (0.36–1.254 μg L^–1^), Pb (0.47–1.24 μg L^–1^), and Cd (0.17–0.40 μg L^–1^), in all samples. The average levels of As (0.70 μg L^–1^) and Pb (0.72 μg L^–1^) were similar, while Cd (0.21 μg L^–1^) was about 3.5 times lower. The toxicological risk assessment considered health hazards from weekly exposure to these metals, providing valuable insights for regulatory toxicology purposes.^55^ In 2017, transition rates of selected metals were determined in various types of teas (*C. sinensis L*. Kuntze) and herbal and fruit infusions.^26^ The content of Al, As, Cd, Cu, Pb, and Hg was determined in both the dry tea and the resulting infusion using ICP–MS. Tea samples were chosen to represent different origins, varieties, leaf grades, and production methods. After microwave-assisted acid digestion of the infusions, the samples were measured by ICP–MS; the following isotopes were monitored: ^27^Al, ^63^Cu, ^75^As, ^111^Cd, ^115^In (internal standard), Pb (average of ^206^Pb, ^207^Pb, and ^208^Pb), and ^202^Hg. Arsenic and Cu were measured using He as a collision gas to prevent isobaric interference caused by adducts that would yield the same *m*/*z*. It provided valuable insights into the metal content of teas and herbal/fruit infusions, with transition rates for the investigated metals varying significantly but generally remaining well below 100%. The study supports the proposal of default transition rates to more accurately estimate exposure levels from the consumption of these products.^26^ A pilot study on trace elements (Al, Cr, Mn, Fe, Co, Ni, Cu, Zn, As, Se, Sr, Cd, Ba, and Pb) in the infusion of medicinal plants used for diabetes treatment was published by Brima and Siddeeg (2022),^27^ also utilizing ICP–MS-based analyses. Five medicinal plants, Tut leaves (*Mulberry*), olive leaves (*O. europaea*), clove (*Syzygium aromaticum*), Luban Dhakar (*Boswellia carterii*), and Karela or bitter melon (*Momordica charantia*), were studied. *M. charantia* showed high levels of essential (Mn, Co, Cu, Se) and toxic (Al, As, Cd, Pb) elements, followed by *S. aromaticum* (Mn) and *B. carterii* (Pb), with some elements exceeding the provisional maximum tolerable daily intake in traditional doses used in diabetes treatments.^27^ Ebrahim et al. (2022)^38^ used an ICP–MS-based methodology to evaluate the health risks of novel and traditional elemental impurities (Ag, Au, Co, Cs, Li, Mo, Se, Sr, and V) in mint tea infusions (*M. piperita L.*) available in Poland. In this study, the infusion samples were analyzed without prior digestion, and the elemental determination was performed using an external calibration curve.^41^

## 6 ICP–MS Hyphenated Techniques for Analysis of Herbal Medicines

### 6.1 LA–ICP–MS

LA–ICP–MS has been established as a powerful analytical technique for chemical investigations of biological and environmental samples. By using a laser to ablate material, this approach enables in situ elemental analysis with high spatial resolution and without requiring extensive sample preparation. Its ability to generate high-resolution images of elemental distribution in plant tissues makes it an essential tool for studies of elemental translocation, elemental toxicity, and the effects of plant exposure to contaminants, providing valuable insights into the absorption and mobility of nutrients and contaminants in plants.^47,48,51,56^ Reproducibility in LA–ICP–MS imaging is driven by sample geometry and calibration; therefore, section thickness (e.g., 10–50 μm) is fixed, substrate/adhesive blanks are verified, and fluence, spot size, and scan speed are kept constant across runs.^47,51^ Quantitative images are obtained using matrix-matched standards with or without internal standardization, and repeatability is evaluated by replicate lines/areas with RSDs reported for key analytes.^47–49^

To investigate the presence of Cd in plant tissues of *Coptis chinensis* Franch., Maciel-Torres et al. (2019)^45^ exposed plants to different concentrations of this metal, showing that bioimaging via LA–ICP–MS elucidates its preferential distribution in the rhizomes, with higher accumulation in the periderm and cortex. This pattern suggests that the outer regions of the plant play a crucial role in Cd accumulation, which could inform mitigation strategies prior to medicinal use. Additionally, the distribution of other elements, such as Ca and P, was correlated to Cd, suggesting interactions that influence metal absorption and transport mechanisms. These findings are fundamental to understanding the response of contaminated plants and developing strategies to minimize Cd transfer to humans via consumption.

Similarly, LA–ICP–MS has been used to investigate the mobility of elements in plant tissues, enabling the mapping of essential nutrients and contaminants. A study by Cui et al. (2023)^47^ on *T. peduncularis* leaves demonstrated quantification of Zn, Cu, Sr, and Mn using matrix-matched gelatin–hydroxypropyl methylcellulose (GA&HPMC) calibration materials with ^13^C internal standardization (Figure 3). The bioimaging revealed distinct distribution patterns for these elements: Zn exhibited a decreasing gradient from the veins to the leaf edges, while Cu was more uniformly distributed with higher concentrations at the leaf base. Strontium predominantly accumulated in the petiole, whereas Mn, although relatively homogeneous, showed higher concentrations in the main vein.

**Figure 3:**
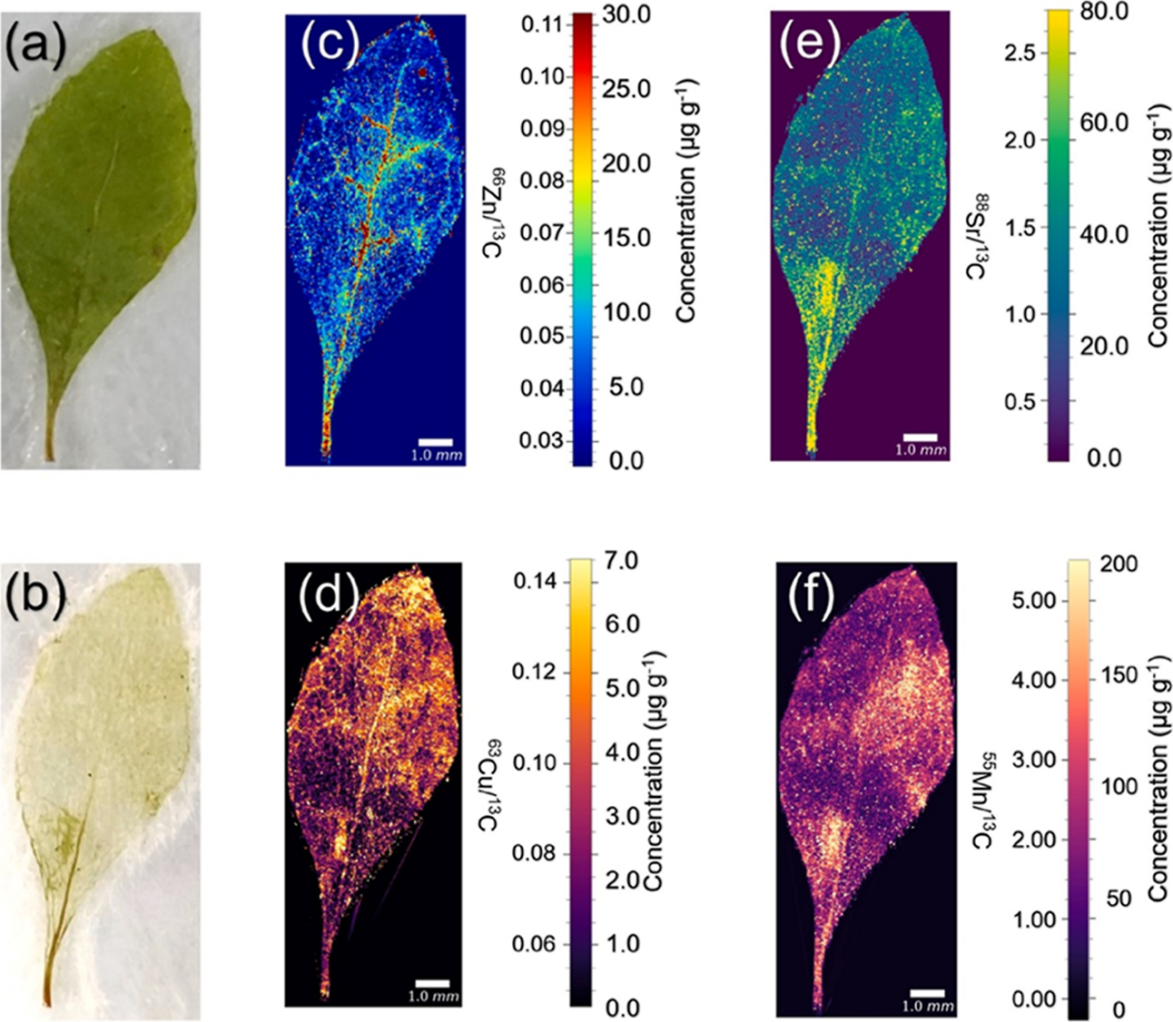
Typical images of elemental distribution in *Triteleia peduncularis* leaf (a) before and (b) after laser ablation; quantitative images of (c) Zn, (d) Cu, (e) Sr, and (f) Mn using ^13^C as an internal standard (photograph courtesy of Anal. Chim. Acta ref 47).

Calibration materials have been widely explored in laser-ablation-based analysis to minimize matrix interferences and potential fractionation effects during ablation. Matrix effects are particularly relevant when analyzing samples of distinct compositions, as they influence the laser–sample interaction and compromise method accuracy. To address this, in addition to the GA&HPMC calibrants used by Cui et al. (2023)^47^ for matrix simulation in calibration, Nunes et al. (2016)^54^ employed filter papers doped with standard solutions. This strategy proved to be effective in reducing matrix effects and was complemented by the use of ^13^C as an internal standard. However, an important limitation to consider is the potential for nonhomogeneous deposition of the standard solution, which may vary with the chosen matrix. Using the proposed calibration materials, As, Cd, Co, Cr, Cu, Mn, Ni, Pb, Sr, V, and Zn were quantified in star anise and passion fruit samples, with LODs ranging from 0.05 to 0.81 μg g^–1^ (As and Mn, respectively) and accuracy (relative errors) below 20%.^48^ To ensure reproducible calibration, homogeneity of doped papers is verified (e.g., replicate spots and line-scan profiles), and acceptance criteria for within-standard relative standard deviation (RSD) are stated; when applicable, background from substrates/adhesives is monitored and blank-corrected.^48,49^

### 6.2 Observations on HPLC–ICP–MS and SEC–ICP–MS

The analysis of herbal medicines using LC–ICP–MS primarily aims to assess the toxicological risks associated with impurities and contaminants in commercially available products and commonly used medicinal plants. The analytical workflow used to analyze medicinal plants enables the determination, separation, and identification of chemical species, using coupled chromatographic methods such as HPLC–ICP–MS and SEC–ICP–MS.^28,29,37,55^ For LC–ICP–MS, reproducibility depends on extraction yield and species stability; therefore, mass balance against totals is assessed, replicate preparations and injections (*n* ≥ 3) are performed, and internal standards as well as collision/reaction cell settings are reported to ensure interlaboratory comparability.^8,15,25,33^ To minimize interconversion, extraction and storage conditions (temperature, time, and oxygen exposure) and filtration pore size are explicitly documented, and where relevant, cold/anaerobic handling is employed.^24,25,33^

A study conducted by Jabłońska-Czapla et al. (2023)^33^ investigated the accumulation of As species in stems and leaves of chamomile (*M. recutita*), peppermint (*Mentha x piperita*), lemon balm (*M. officinalis*), and sage (*S. officinalis*) grown in contaminated soil. The analysis was performed using HPLC–ICP–MS, with an ion-exchange column, which efficiently separated various As species including As­(III), As­(V), arsenobetaine (AB), monomethylarsonic acid (MMA), and dimethylarsinic acid (DMA). The gradient elution used ammonium nitrate buffers at pH 8.7, allowing baseline resolution of all As species. For As extraction from plant samples, 1 g of plant material was treated with 10 mL of a water/methanol solution (1:9 ratio) and shaken for 2 h. After being filtered through a 0.22 μm syringe filter, the extracts were injected into the HPLC–ICP–MS system for As speciation analysis. The results showed that peppermint accumulated the highest As levels, particularly those of organoarsenical species. These findings highlight the effectiveness of HPLC–ICP–MS in accurately assessing As contamination and speciation in medicinal plants. In such applications, reporting LOD/LOQ calculations, recovery of spiked species, and between-preparation RSDs strengthens conclusions on speciation accuracy and reproducibility.^24,25,33^

SEC–ICP–MS was employed by Ebrahim et al. (2020)^37^ to assess Zn species content and protein profiles in medicinal plants traditionally applied in diabetes treatment. The plants analyzed in the study included sesame seeds (*S. indicum*), olive leaves (*O. europaea*), bilberry fruit (*V. myrtillus*), black cumin seeds (*N. sativa*), fenugreek seeds (*T. foenum-graecum*), pearl millet seeds (*Pennisetum glaucum*), carthage whole plant (*Calligonum comosum*), colocynth fruit (*Citrullus colocynthis*), bitter melon fruit (*M. charantia*), barbary Figure fruit (*Opuntia ficus-indica*), and ramth whole plant (*Haloxylon salicornicum*). Zinc-protein profiles were analyzed by SEC–ICP–MS. The plants were subjected to extraction with water, and the mixture was centrifuged and filtered before analysis. Two columns were used to achieve separation of compounds with high molecular mass (1–700 kDa) and low molecular mass (<2 kDa). Isocratic elution was employed using a mixture of ammonium acetate and methanol; the eluate from the columns passed through a UV detector (λ = 254 and 220 nm) and was introduced into the ICP–MS via a Meinhard nebulizer and cyclone spray chamber. Dynamic Reaction Cell mode with NH_3_ as the reaction gas was used to eliminate isobaric interferences, and the isotope ^66^Zn was monitored. The results showed that the highest Zn concentrations were found in the 50–60 and 70–87 kDa protein fractions, with certain plant species, such as *M. charantia* and *C. colocynthis*, exhibiting the highest extractable Zn concentrations. This study highlights SEC–ICP–MS as an effective technique for profiling Zn distribution in medicinal plants, providing insights into their potential for diabetes treatment through their Zn content.^37^ For SEC–ICP–MS, reproducibility is supported by replicate extractions/injections, calibration of retention time with protein standards, and reporting of within-fraction RSDs; UV traces acquired in parallel assist fraction assignment and quality control.

SEC–ICP–MS was employed to measure Cr and Mn in the protein fractions of plants applied in traditional antidiabetes treatments.^38^ The samples were subjected to extraction with hot water (80 °C), simulating the typical use of the plants as tea. The extracts were then analyzed by SEC–ICP–MS using an isocratic elution with a 9:1 mixture of 50 mmol L^–1^ ammonium acetate (pH 5.8) and 500 mmol L^–1^ ammonium acetate, 10 mmol L^–1^ tris­(hydroxymethyl)­aminomethane, and 5% (v v^–1^) methanol (pH 8.0). The separation enabled quantification of Cr and Mn bound to 11 protein fractions. Chromium was determined in all SEC fractions (primarily protein fractions), except for the 1.9–3.7 kDa range, while Mn was found in all SEC fractions, excluding the 100–120 and 1.3–3.7 kDa fractions. However, the highest concentrations of available Cr species were found in the 10–14 and 0.05–0.40 kDa fractions, while Mn was predominantly bound to the 0.05–0.40 kDa fraction.^38^ Because hot-water extraction may alter coordination or oxidation states, stability checks (e.g., time-course at 4–8 °C versus room temperature) and recovery of fortified species are documented to support interpretation of fraction-bound metals.^24,25,38^

Overall, LC–ICP–MS and speciation methods provide valuable results for herbal medicines, enabling the precise identification and quantification of trace-element species. These methods reveal important insights into elemental distributions and chemical speciation, contributing to safety and quality assessments of herbal medicines. The ability to separate and quantify distinct species enhances the accuracy and reliability of medicinal plant evaluations, thereby supporting their therapeutic potential. Finally, transparent QA/QC (procedural blanks, spike recoveries, and CRMs when available) and explicit precision targets enable critical assessment of method reproducibility across studies.

## 7 State of the Art and Outlook: Methods and Trends

Beyond the conventional solution ICP–MS and the hyphenated approaches already discussed, complementary tools are routinely employed depending on the analytical question. Techniques such as ICP OES and TXRF/XRF provide rapid screening for multielement profiles with simpler sample handling; HR–CS GF AAS offers sensitivity for selected elements in small aliquots; LC–HRMS and NMR address organic markers and authenticity; and IRMS/radiocarbon (^14^C) supports source attribution. For solid samples, LA–ICP–MS enables spatially resolved mapping,^47–49^ while Raman/FT-IR assists in identifying inorganic adulterants or matrices. These methods are applied orthogonally to confirm findings, resolve spectral or matrix interferences, and reduce uncertainty in complex herbal matrices.

Looking ahead, interference control and imaging throughput continue to advance: ICP–MS/MS (triple-quadrupole ICP–MS) provides cleaner interference removal;^57^ single-particle ICP–MS (sp–ICP–MS) and single-cell ICP–MS (sc–ICP–MS) enable nanoparticulate and cellular-scale assessments;^58,59^ and LA–ICP–TOF imaging supported by improved calibration strategies accelerates multielement mapping.^60,61^ Concurrent progress in green sample preparation (low-acid microwave digestion, microextraction, and automation), matrix-matched and digitally printed/microdroplet calibrants for LA, and machine learning/chemometrics for classification is expected to improve robustness and comparability.^40,44,60,61^ Interlaboratory studies, plant-based CRMs, and transparent QA/QC reporting remain priorities to strengthen reproducibility across studies.^9,24,25,32^

## 8 Conclusions

Owing to its high sensitivity, low detection limits, elemental selectivity, isotopic-ratio capability, and wide linear dynamic range, ICP–MS remains a leading technique for determining essential and toxic elements in plants, especially in herbal medicines. Studies focused on medicinal plants face several key challenges, including complex sample preparation, low concentrations of trace elements, detection-limit constraints, and matrix effects in multielement analyses, all of which can affect the precision and reliability of results. Additionally, coupling ICP–MS with complementary analytical techniques, such as LA–ICP–MS, LC–ICP–MS, HPLC–ICP–MS, and FI–CVG–ICP–MS, greatly enhances the analytical capability for the comprehensive analysis of herbal medicines. These coupled techniques enable elemental determination, high-resolution profiling of elemental composition, and chemical speciation analysis. As a result, they facilitate a comprehensive understanding of the chemical, nutritional, and pharmacological properties of herbal medicines, thereby improving their quality control, safety assessment, and evaluation of their therapeutic potential. Ensuring reproducibility in herbal matrices requires standardized sample preparation and transparent QA/QC; interlaboratory comparisons and validated matrix-matched calibration remain the key to comparability. In practical terms, (i) bulk ICP–MS should employ closed-vessel microwave digestion with diluted HNO_3_/H_2_O_2_ and maintain postdigestion TDS ≤ 0.2% m/v, with internal standardization (e.g., In/Rh/Ir) and CRC operation, and trueness verified using plant-based CRMs and spike recoveries; (ii) LC–/SEC–ICP–MS should report extraction mass balance, species-stability checks, and replicate preparations/injections; and (iii) LA–ICP–MS should use constant section thickness and matrix-matched calibrants (e.g., doped papers), with quantitative imaging supported by replicate lines/areas and RSD reporting. For regulators and standard-setters, harmonized reporting (sample masses, digestion programs, TDS, internal standards/CRC settings), minimum QA/QC acceptance criteria (e.g., recoveries 80–120%; precision ≤15% at ultratrace), and interlaboratory trials are recommended to strengthen comparability and risk assessment for herbal products.

## References

[1] Thomas, R. A beginner’s guide to ICP-MS – Part I; Spectroscopy, 2001; Vol. 16, pp 38–42.

[2] Thomas, R. Practical Guide to ICP-MS and Other Atomic Spectroscopy Techniques, A Tutorial for Beginners, 4th ed.; CRC Press: Boca Raton, FL, 2023; pp 1–460.

[3] Ahmad R., Shaaban H., Issa S. Y., Alsaad A., Alghamdi M., Hamid N., Osama R., Algarni S., Mostafa A., Alqarni A. M. (2022). ICP-MS determination of elemental abundance
in traditional medicinal plants commonly used in the Kingdom of Saudi
Arabia. Food Addit. Contam.:Part B.

[4] Chen W., Yang Y., Fu K., Zhang D., Wang Z. (2022). Progress in
ICP-MS analysis of minerals and heavy metals in traditional medicine. Front. Pharmacol.

[5] Oliveira A. P., Naozuka J., Landero-Figueroa J. A. (2025). Selenium
speciation in enriched *Pleurotus* mushrooms by mixed-mode
HPLC-ICP-MS. J. Food Compos. Anal..

[6] Rawat H., Bhat S. A., Dhanjal D. S., Singh R., Gandhi Y., Mishra S. K., Kumar V., Shakya S. K., Narasimhaji C. V., Singh A., Singh R., Acharya R. (2024). Emerging techniques
for the trace elemental analysis of plants and food-based extracts:
A comprehensive review. Talanta Open.

[7] Wiggenhauser M., Moore R. E. T., Wang P., Bienert G. P., Laursen K. H., Blotevogel S. (2022). Stable isotope fractionation of metals
and metalloids
in plants: a review. Front. Plant Sci..

[8] Linge K. L., Jarvis K. E. (2009). Quadrupole ICP-MS: Introduction to
instrumentation,
measurement techniques and analytical capabilities. Geostand. Geoanal. Res..

[9] Krug, F. J. ; Rocha, F. R. P. Métodos de preparo de amostras para análise elementar; EDIT-SBQ: São Paulo, 2016; pp 1–572. ISBN: 978–85–64099–18–0.

[10] Thomas, R. A beginner’s guide to ICP-MS – Part III: The plasma source; Spectroscopy, 2001; Vol. 16, pp 26–30.

[11] Thomas, R. A beginner’s guide to ICP-MS – Part II: The sample-introduction system; Spectroscopy, 2001; Vol. 16, pp 56–60.

[12] Thomas, R. A beginner’s guide to ICP-MS – Part IV: The interface region; Spectroscopy, 2001; Vol. 16, pp 26–30.

[13] Thomas, R. A beginner’s guide to ICP-MS – Part V: The ion focusing system; Spectroscopy, 2001; Vol. 16, pp 38–42.

[14] Thomas, R. A beginner’s guide to ICP-MS – Part VI: The mass analyser; Spectroscopy, 2001; Vol. 16, pp 44–48.

[15] Bolea-Fernandez E., Balcaen L., Resano M., Vanhaecke F. (2017). Overcoming
spectral overlap via inductively coupled plasma-tandem mass spectrometry
(ICP-MS/MS). A tutorial review. J. Anal. At.
Spectrom..

[16] Thomas, R. A beginner’s guide to ICP-MS – Part IX: Mass separation: collision/reaction cell technology; Spectroscopy, 2002; Vol. 17, pp 42–48.

[17] Thomas, R. A beginner’s guide to ICP-MS – Part VII: Mass separation devices – double-focusing magnetic technology; Spectroscopy, 2001; Vol. 16, pp 22–26.

[18] AlChoubassi G., Aszyk J., Pisarek P., Bierla K., Ouerdane L., Szpunar J., Lobinski R. (2018). Advances in mass spectrometry for
iron speciation in plants. TrAC Trends Anal.
Chem..

[19] Fu L., Shi S.-Y., Chen X.-Q. (2018). Accurate
quantification of toxic
elements in medicine food homologous plants using ICP-MS/MS. Food Chem..

[20] Thomas, R. A beginner’s guide to ICP-MS – Part VIII: Mass separation: time-of-flight technology; Spectroscopy, 2002; Vol. 17, pp 36–40.

[21] Corte-Rodríguez M., Álvarez-Fernández R., García-Cancela P., Montes-Bayón M., Bettmer J. (2020). Single-cell ICP-MS using online sample
introduction systems: current developments and remaining challenges. TrAC, Trends Anal. Chem..

[22] Mokgalaka N. S., Gardea-Torresdey J. L. (2006). Laser ablation
inductively coupled plasma mass spectrometry:
principles and applications. Appl. Spectrosc.
Rev..

[23] Pozebon D., Scheffler G. L., Dressler V. L. (2017). Recent applications of laser ablation
inductively coupled plasma mass spectrometry (LA-ICP-MS) for biological
sample analysis: a follow-up review. J. Anal.
At. Spectrom..

[24] Wang T. (2007). Liquid chromatography–inductively
coupled plasma mass spectrometry (LC–ICP–MS). J. Liq. Chromatogr. Relat. Technol..

[25] Clough R., Harrington C. F., Hill S. J., Madrid Y., Tyson J. F. (2018). Atomic
spectrometry update: review of advances in elemental speciation. J. Anal. At. Spectrom..

[26] Schulzki G., Nüßlein B., Sievers H. (2017). Transition rates of selected metals
determined in various types of teas (*Camellia sinensis* L. Kuntze) and herbal/fruit infusions. Food
Chem..

[27] Brima E. I., Siddeeg S. M. (2022). Pilot study of trace
elements in the infusion of medicinal
plants used for diabetes treatment. Int. J.
Anal. Chem..

[28] Girme A., Saste G., Balasubramaniam A. K., Pawar S., Ghule C., Hingorani L. (2020). Assessment
of *Curcuma longa* extract
for adulteration with synthetic curcumin by analytical investigations. J. Pharm. Biomed. Anal..

[29] Deng C., Tang Q., Yang Z., Dai Z., Cheng C., Xu Y., Chen X., Zhang X., Su J. (2022). Effects of iron oxide
nanoparticles on phenotype and metabolite changes in hemp clones (*Cannabis sativa* L.). Front. Environ.
Sci. Eng..

[30] Filipiak-Szok A., Kurzawa M., Szłyk E. (2015). Determination
of toxic metals by
ICP-MS in Asiatic and European medicinal plants and dietary supplements. J. Trace Elem. Med. Biol..

[31] de
Oliveira N. M., Lopes L., Chéu M. H., Soares E., Meireles D., Machado J. (2023). Updated mineral composition
and potential therapeutic properties of different varieties of olive
leaves from *Olea europaea*. Plants.

[32] Qazimi B., Stafilov T., Andonovska K. B., Tašev K., Geskovski N., Dragusha S., Koraqi H., Ejupi V. (2024). Characterization
of mineral composition of leaves and flowers of wild-growing *Sambucus nigra*. Acta Pharm..

[33] Jabłońska-Czapla M., Michalski R., Nocoń K., Grygoyć K. (2019). The mobility
of arsenic and its species in selected herbs. Arch. Environ. Prot..

[34] Dinda B., Dinda M., Dinda S., Ghosh P. S., Das S. K. (2024). Anti-SARS-CoV-2,
antioxidant and immunomodulatory potential of dietary flavonol quercetin:
focus on molecular targets and clinical efficacy. Eur. J. Med. Chem. Rep.

[35] Nicoliche T., Bartolomeo C. S., Lemes R. M. R., Pereira G. C., Nunes T. A., Oliveira R. B., Nicastro A. L. M., Soares É. N., da Cunha Lima B. F., Rodrigues B. M. (2024). Antiviral, anti-inflammatory
and antioxidant effects of curcumin and curcuminoids in SH-SY5Y cells
infected by SARS-CoV-2. Sci. Rep..

[36] Rajagopal K., Varakumar P., Baliwada A., Byran G. (2020). Activity of
phytochemical
constituents of *Curcuma longa* (turmeric) and *Andrographis paniculata* against coronavirus (COVID-19):
an in silico approach. Futur. J. Pharm. Sci..

[37] Ebrahim A. M., Alnajjar A. O., Mohammed M. E., Idris A. M., Mohammed M. E., Michalke B. (2020). Investigation of total zinc contents
and zinc-protein
profile in medicinal plants traditionally used for diabetes treatment. BioMetals.

[38] Ebrahim A. M., Idris A. M., Alnajjar A. O., Michalke B. (2020). Cr and Mn total, accessible
species, and protein-fraction contents in plants used for traditional
anti-diabetes treatment. J. Trace Elem. Med.
Biol..

[39] Gaiad J. E., Hidalgo M. J., Villafañe R. N., Marchevsky E. J., Pellerano R. G. (2016). Tracing the geographical origin of
Argentinean lemon
juices based on trace element profiles using advanced chemometric
techniques. Microchem. J..

[40] Muller A. L., Muller E. I., Barin J. S., Flores E. M. M. (2015). Microwave-assisted
digestion using diluted acids for toxic element determination in medicinal
plants by ICP-MS in compliance with United States Pharmacopeia requirements. Anal. Methods.

[41] Milan J., Frydrych A., Noga M., Kondratowicz-Pietruszka E., Krośniak M., Jurowski K. (2022). The control of novel and traditional
elemental impurities: Ag, Au, Co, Cs, Li, Mo, Se, Sr, and V in mint
tea infusions (peppermint, *Mentha piperita* L.) available
in Poland: A health risk assessment. Int. J.
Environ. Res. Public Health.

[42] Jurowski K., Kondratowicz-Pietruszka E., Krośniak M. (2023). The toxicological
safety assessment of heavy metal impurities (As, Pb, and Cd) in mint
tea infusions (*Mentha piperita* L.) available in Polish
markets. Biol. Trace Elem. Res..

[43] Turan D., Kocahakimoglu C., Kavcar P., Gaygısız H., Atatanir L., Turgut C., Sofuoğlu S. C. (2011). The use
of olive tree (*Olea europaea* L.) leaves as a bioindicator
for environmental pollution in the Province of Aydın, Turkey. Environ. Sci. Pollut. Res..

[44] Shuai M., Peng C., Yang Y., Ren Y., Hou R., Cao L., Ning J., Cai H. (2023). Characterization of
elements and
carbon and nitrogen stable isotopes in American ginseng (Panax quinquefolius
L.): determining the geographical origin combining with chemometrics. J. Food Compos. Anal..

[45] Maciel-Torres S. P., Jacobo-Salcedo M. d. R., Figueroa-Viramontes U., Pedroza-Sandoval A., Trejo-Calzada R., Rivas-García T. (2024). Evaluation of the arsenic extraction
capacity of *Ricinus communis*. L. Terra Latinoam..

[46] Rehman H. U., Hassan N. U., Jelani M., Alanazi K. D., Ahmed N., Ullah T. S., Javed M. S., Fawy K. F. (2024). Compositional
analysis
of *Swertia chirayita* medicinal plant using laser-induced
breakdown spectroscopy and ICP-MS. PLoS One.

[47] Cui Z., He M., Chen B., Hu B. (2023). In-situ elemental quantitative imaging
in plant leaves by LA-ICP-MS with matrix-matching external calibration. Anal. Chim. Acta.

[48] Pedrosa
Diniz A., Rodrigues Kozovits A., De Carvalho Lana C., Trópia De Abreu A., Garcia Praça Leite M. (2019). Quantitative
analysis of plant leaf elements using the LA-ICP-MS technique. Int. J. Mass Spectrom..

[49] Nunes M. A. G., Voss M., Corazza G., Flores E. M. M., Dressler V. L. (2016). External
calibration strategy for trace element quantification in botanical
samples by LA-ICP-MS using filter paper. Anal.
Chim. Acta.

[50] Zhou J., Guo W., Jin L., Hu S. (2022). Elemental analysis of solid food
materials using a reliable laser ablation inductively coupled plasma
mass spectrometry method. J. Agric. Food Chem..

[51] Huang W., Bai Z., Jiao J., Yuan H., Bao Z., Chen S., Ding M., Liang Z. (2019). Distribution and chemical
forms of
cadmium in *Coptis chinensis* Franch. determined by
laser ablation ICP-MS, cell fractionation, and sequential extraction. Ecotoxicol. Environ. Saf..

[52] Borisov O. V., Bannochie C. J., Russo R. E. (2001). Laser ablation inductively
coupled
plasma mass spectrometry of pressed pellet surrogates for Pu materials
disposition. Appl. Spectrosc..

[53] You H., Gershon H., Goren F., Xue F., Kantowski T., Monheit L. (2022). Analytical strategies to determine the labelling accuracy
and economically-motivated adulteration of “natural”
dietary supplements in the marketplace: Turmeric case study. Food Chem..

[54] Forsyth J. E., Nurunnahar S., Islam S. S., Baker M., Yeasmin D., Islam M. S., Rahman M., Fendorf S., Ardoin N. M., Winch P. J., Luby S. P. (2019). Turmeric means “yellow”
in Bengali: Lead chromate pigments added to turmeric threaten public
health across Bangladesh. Environ. Res..

[55] Jurowski K., Krośniak M. (2023). The toxicological
risk assessment (TRA) of total chromium
impurities in *Menthae piperitae tinctura* (*Mentha × piperita* L., folium) available in Polish pharmacies
including regulatory approaches with special emphasis of Cr speciation
and genotoxicity. Biol. Trace Elem. Res..

[56] Becker J. S., Matusch A., Wu B. (2014). Bioimaging
mass spectrometry of trace
elements – recent advance and applications of LA-ICP-MS: a
review. Anal. Chim. Acta.

[57] Ma Q., Yang Z., Yang Y., Chu Z. (2023). Trace-Element Analysis
and Radiometric Dating by Inductively Coupled Plasma–Tandem
Mass Spectrometry: Approaches and Applications to Metallogeny. Ore Geol. Rev..

[58] Laycock A., Clark N. J., Clough R., Smith R., Handy R. D. (2022). Determination
of Metallic Nanoparticles in Biological Samples by Single Particle
ICP-MS: A Systematic Review from Sample Collection to Analysis. Environ. Sci.: Nano.

[59] Silva A. B. S., Arruda M. A. Z. (2023). Single-Cell ICP-MS
to Address the Role of Trace Elements
at a Cellular Level. J. Trace Elem. Med. Biol..

[60] Mervič K., Šala M., Theiner S. (2024). Calibration Approaches
in Laser Ablation
Inductively Coupled Plasma Mass Spectrometry for Bioimaging Applications. TrAC, Trends Anal. Chem..

[61] Schweikert A., Theiner S., Wernitznig D., Schoeberl A., Schaier M., Neumayer S., Keppler B. K., Koellensperger G. (2022). Micro-Droplet-Based
Calibration for Quantitative Elemental Bioimaging by LA-ICPMS. Anal. Bioanal. Chem..

